# Circulation of Different Lineages of Dengue Virus 2, Genotype American/Asian in Brazil: Dynamics and Molecular and Phylogenetic Characterization

**DOI:** 10.1371/journal.pone.0059422

**Published:** 2013-03-22

**Authors:** Betânia Paiva Drumond, Adriano Mondini, Diane J. Schmidt, Roberta Vieira de Morais Bronzoni, Irene Bosch, Maurício Lacerda Nogueira

**Affiliations:** 1 Laboratório de Virologia, Universidade Federal de Juiz de Fora, Juiz de Fora, Minas Gerais, Brazil; 2 Laboratório de Saúde Pública, Faculdade de Ciências Farmacêuticas, Universidade Estadual Paulista, Araraquara, São Paulo, Brazil; 3 Department of Infectious Disease and Global Health, Cummings School of Veterinary Medicine, Tufts University, North Grafton, Massachusetts, United States of America; 4 Universidade Federal do Mato Grosso, Sinop, Mato Grosso, Brazil; 5 Genome Resources in Dengue Consortium, Massachusetts Institute of Technology. Cambridge, Massachusetts, United States of America; 6 Laboratório de Pesquisa em Virologia, Faculdade de Medicina de São José do Rio Preto (FAMERP), São José do Rio Preto, São Paulo, Brazil; University of Texas Medical Branch, United States of America

## Abstract

The American/Asian genotype of Dengue virus type 2 (DENV-2) was introduced into the Americas in the 80′s. Although there is no data showing when this genotype was first introduced into Brazil, it was first detected in Brazil in 1990. After which the virus spread throughout the country and major epidemics occurred in 1998, 2007/08 and 2010. In this study we sequenced 12 DENV-2 genomes obtained from serum samples of patients with dengue fever residing in São José do Rio Preto, São Paulo (SJRP/SP), Brazil, in 2008. The whole open reading frame or envelope sequences were used to perform phylogenetic, phylogeographic and evolutionary analyses. Isolates from SJRP/SP were grouped within one lineage (BR3) close to isolates from Rio de Janeiro, Brazil. Isolates from SJRP were probably introduced there at least in 2007, prior to its detection in the 2008 outbreak. DENV-2 circulation in Brazil is characterized by the introduction, displacement and circulation of three well-defined lineages in different times, most probably from the Caribbean. Thirty-seven unique amino acid substitutions were observed among the lineages, including seven amino acid differences in domains I to III of the envelope protein. Moreover, we dated here, for the first time, the introduction of American/Asian genotype into Brazil (lineage BR1) to 1988/89, followed by the introduction of lineages BR2 (1998–2000) and BR3 (2003–05). Our results show a delay between the introduction and detection of DENV-2 lineages in Brazil, reinforcing the importance and need for surveillance programs to detect and trace the evolution of these viruses. Additionally, Brazilian DENV-2 differed in genetic diversity, date of introduction and geographic origin and distribution in Brazil, and these are important factors for the evolution, dynamics and control of dengue.

## Introduction

The four serotypes of dengue virus (DENV 1–4) (family *Flaviviridae*, genus *Flavivirus*) are antigenically and genetically distinct. DENV has a single-stranded positive-sense RNA genome of 10,700 nucleotides surrounded by a nucleocapsid and covered by a lipid envelope containing viral glycoproteins. The RNA genome contains a single open reading frame that encodes a precursor polyprotein, which is co- and post-translationally cleaved into three structural (C, prM and E) and seven nonstructural (NS1, NS2A, NS2B, NS3, NS4A, NS4B, NS5) proteins. Infection by any DENV serotype can cause an acute self-limited febrile illness, the classic dengue fever (DF) and/or severe syndromes characterized by hemorrhage and capillary leakage, dengue hemorrhagic fever and dengue shock syndrome (DHF/DSS), according to the old classification system, which is has been recategorized by the World Health Organization into dengue with or without warning signs and severe [1]–[3]. It is estimated that 2.5 to 3 billion people are at risk of DENV infection in more than 100 countries with about 100 million infections and up to 250,000 cases of DHF/DSS worldwide each year [4].

Phylogenetic analyses of dengue virus type 2 (DENV-2) based on the envelope gene has revealed the existence of six genotypes: (*i*) Asian I, (*ii*) Asian II, (*iii*) Cosmopolitan, (*iv*) American, (*v*) American/Asian and (*vi*) sylvatic [5]–[7]. The Asian/American genotype groups strains from Southeast Asia in one subclade and clusters strains from the Caribbean region and Latin America countries into a second subclade [5], [6]. Following the introduction of the American/Asian genotype to the American continent, likely from Vietnam into Cuba, the first case of Dengue hemorrhagic fever was reported in the Americas in 1981 [8]. Since then, the American/Asian genotype has been responsible for the displacement of less virulent strains of the American genotype from many regions of the Americas and also caused major epidemics with increased pathogenicity on this continent [9]–[11].

DENV-1 was probably introduced into Brazil in the 80′s [12]–[13] with the first cases being reported in 1981 in the North region and later, in 1986 in Rio de Janeiro, Southeast region. DENV-2 was detected in 1990, in Rio de Janeiro [14], but there is no clear data to indicate when the American/Asian genotype was introduced into Brazil [15]. Sometime between 1997 and 2000, DENV-3 was likely introduced into the country [16] and the first cases were reported in 2000, in Rio de Janeiro [14]. DENV-4 was probably introduced into the country in 2004–2006 [17], as suggested by previous isolates in 2005, in the North region [18] and reemerged in Brazil in 2010–11 [17].

Since the first detection of DENV-2, in Brazil, in 1990, it has been isolated in 24 of the 26 states in the country. Moreover, an increase in both the number of epidemics and disease severity has been observed since its introduction [14]; [19]–[20]. DENV-2 has caused major epidemics in Brazil in 1998, 2007/08, and in 2010. In 2010 two different lineages of DENV-2 belonging to the American/Asian genotype were responsible for epidemics in the country [21]–[22]. According to the Pan American Health Organization [23]–[24], approximately 1.3 million cases of dengue were reported in the Brazil in 2007 and 2008. More than 17,400 cases of severe dengue and 5,700 cases of DHF were reported during this period. Six hundred people died from dengue infection in this two-year period [25]–[26]. Forty per cent of the total cases took place in the Southeast region, comprising the states of São Paulo, Rio de Janeiro, Minas Gerais and Espírito Santo [25]–[26]. Additionally, 15% of total cases from Brazil in those years, were concentrated in São Paulo State (more than 104,000 cases of DF, 339 cases of severe dengue, 80 cases of DHF, 12 cases of DSS and 27 deaths) [27]. São José do Rio Preto (SJRP), which is a city located in the northwestern portion of São Paulo state - with a total area of 431 Km^2^ and an urban area of 119.5 km^2^, an estimated population of 414,272 inhabitants for 2008–presented with more than 9,500 cases of DF, 40 cases of severe dengue, four cases of DHF and two deaths caused by DENV infection [28]–[29], during the years of 2007 and 2008. DENV-1 transmission was first reported in SJRP in the early 1990s. Since then, dengue cases have been reported every year in the city and DENV-2 and DENV-3 were first introduced in 1998 and 2005 respectively [30]. DENV-2 has been circulating since the beginning of the decade in lower or higher frequencies, despite the major circulation of other serotypes.

In this study, we sequenced 12 DENV-2 genomes obtained from serum samples of patients with dengue fever, from SJRP, in 2008, and characterize them at the molecular phylogeographic and phylogenetic levels. These sequences were compared to sequences from different genotypes, including sequences from Brazilian DENV-2 isolates. Analyses were performed to estimate the degree of genetic variability of DENV-2 in SJRP and Brazil, to trace the amino acid substitutions in the deduced amino acid sequences of the entire open reading frame (ORF) and to check the selection pressures acting on these isolates. Phylogenetic analyses based on Maximum likelihood and Bayesian methods as well as coalescent and phylogeographic analyses were performed (using the entire ORF and/or envelope sequences) to infer the origin and phylogenetic relationship of these isolates and to trace the time of introduction of DENV-2 lineages into the country, in order to gain insights into the dynamics of DENV-2 circulation in Brazil as well its genetic diversity. Our results demonstrated the existence of three well-supported DENV-2 lineages in Brazil differing in genetic diversity, year of introduction, geographic origin and distribution.

## Materials and Methods

### Strain and Sample Preparation

The project was approved by the Review Board of Faculdade de Medicina de São José do Rio Preto (Protocol 6312/2005) and blood collection was performed with the written informed consent of the patients. The strains DENV-2/BR/BID-V3653/2008, DENV-2/BR/BID-V3650/2008, DENV-2/BR/BID-V3638/2008, DENV-2/BR/BID-V3481/2008, DENV-2/BR/BID-V3495/2008, DENV-2/BR/BID-V3640/2008, DENV-2/BR/BID-V3645/2008, DENV-2/BR/BID-V3486/2008, DENV-2/BR/BID-V3483/2008, DENV-2/BR/BID-V3637/2008, DENV-2/BR/BID-V3648/2008 and DENV-2/BR/BID-V3644/2008 (GenBank accession numbers GU131885, GU131884, GU131879, GQ868549, GU131864, GU131880, GU131882, GQ868551, GQ868550, HM181971, GU131883, GU131881) were obtained from patients that sought medical care in health facilities in SJRP/SP, Brazil, in 2008. One hundred and forty µl of serum were used for RNA extraction using QiAmp Viral RNA kit (QIAGEN).

### cDNA Synthesis and Sequencing

The cDNA was produced with a 20 µl reverse transcription reaction containing: 1ul Superscript III Reverse Transcriptase (Invitrogen); random hexamers (1 µl of 50 ng/µl stock); specific 3′ reverse primer (1 µl of 10 µM stock) and 5 µl of template RNA. The 5′ primer was used for specific priming of the RT reaction of samples of DENV-2. Twenty microliters of viral cDNA were diluted in 800 µl of water, as template for 96 specific PCR reactions. The 10 µl PCR reaction contained 3 µl of template; 0.03 µl of pfuUltra II polymerase 1 (5 U/µl) (Stratagene); 100 mM dNTPs (Applied Biosystems) and 4 µl of a mixture of forward and reverse primers (0.5 µM stock) (Table S1). The primers were synthesized with M13 sequence tags so that PCR amplicons could be sequenced using universal M13 forward and reverse primers. The PCR reactions produced 96 overlapping amplicons, each 500–900 nucleotides (nt) in length, which were subsequently sequenced bidirectionally using the Big Dye chemistry on ABI3730xl DNA sequencers according to Applied Biosystems protocols, following the same strategy used for DENV-3 [31] and DENV-1 [13].

### Data Sets

The 91 DENV-2 genome sequences used in our analyses were selected first by retrieving all the DENV-2 complete genome sequences available in GenBank (April, 2012) and aligning them using MAFFT [32]. A neighbor-joining tree was then constructed using Mega 5.0 [33]. We then chose to include all the unique Brazilian DENV-2 complete sequences (24 genomes) and selected closely related isolates in the same or neighboring clades, in addition we included sequences from different decades and finally we selected reference sequences from the major genotypes; Asian I, Asian II, Cosmopolitan, American and American/Asian. All sequences were comprised of the whole coding region of the DENV-2 genome [10,173 nt, corresponding to the open reading frame (ORF), from the first nt of the anchored capsid protein gene to the last nt of the NS5 gene] (Table S2). Additionally, all Brazilian DENV-2 sequences of the envelope protein (E) gene were retrieved from the nucleotide database (with the exception of 11 sequences that contained incomplete information regarding geographic origin within Brazil). The E gene sequences were added to the first alignment and used to create an alignment of the E gene, containing a total of 144 sequences (77 sequences from Brazilian isolates) of 1495 nt (Table S2). For selection analyses, another data set was created with 100 ORF sequences from DENV-2 American/Asian genotype (Table S2). This dataset contained 21 Brazilian DENV-2 sequences including 8 sequence isolates from SJRP, excluding identical sequences and based on the first neighbor-joining tree, other sequences from American/Asia genotypes were also selected as described (based on the NJ tree constructed above). The nucleotide substitution model for each data set was chosen using ModelTest server [34].

### Phylogenetic Analyses

Maximum Likelihood trees were constructed using the alignment containing 91 DENV-2 complete ORF sequences using the General Time Reversible model and Gamma distribution (GTR +*G*) implemented in MEGA 5.0 [33]. Rates of variation among sites were estimated for each dataset and four discrete Gamma categories were used to model evolutionary rate differences among sites. The reliability of branching patterns was tested through 1000 bootstrap sampling. To include a greater number of Brazilian sequences, phylogenetic analyses were also performed using the alignment containing 144 sequences of the DENV-2 E gene. Using this later data set, Maximum likelihood trees were reconstructed using MEGA 5.0 [33] as previously described, using the Tamura Nei model and Gamma distribution (TN93+ *G*).

### Genetic Diversity and Selection Analyses

Estimates of the evolutionary divergence within and among different lineages were calculated based on 77 envelope sequences of Brazilian DENV-2 isolates. The mean distance, given by the number of base substitutions per site from all sequence pairs within or among different lineages was computed using the Tamura-Nei model implemented in Mega 5.0 [33] and values were further converted into percentage. The HyPhy package in Datamonkey webserver (www.datamonkey.org) [35] was used to check the selection pressures on the codons of the entire ORF of DENV-2, using different methods: single-likelihood ancestor (SLAC), fixed–effects likelihoods (FEL), internal branch fixed-effects likelihood (IFEL) and FUBAR. The GTR model was used and significance levels of p<0.05 (SLAC, FEL, IFEL), or posterior probability >0.9 (FUBAR) were chosen for the analyses.

### Bayesian Coalescent and Discrete Phylogeographic Analyses

Time of the most recent common ancestor (MRCA) for some lineages was estimated using BEAST package v.1.6.1 with Markov Chain Monte Carlo algorithms [36]. Input files for BEAST were created with BEAUTi graphical interface [37] based on 144 sequences of the E gene, including sequences from all genotypes, excepting the sylvatic genotype (Table S2), and a total of 77 Brazilian sequences. The calibration point was the year each strain was isolated/obtained. Runs were performed using the Bayesian Skyline plot, under strict or relaxed (uncorrelated lognormal) molecular clock, and using the estimated rate of 7.50×10^−4^ substitutions per site, per year, as previously described [38]. One hundred million chains were run and the first 10 million steps were discarded. Convergence of parameters was verified with Tracer v.1.5.0 [39] and uncertainties were addressed as the 95% highest probability density (HPD) intervals. The trees were sampled at each 5,000 steps, resulting in 18,000 trees, which were summarized in a maximum clade credibility tree using TreeAnotator v.1.6.1 [40] that was then visualized in FigTree v.1.3.1 [41]. This analysis was also carried out using the smaller dataset, containing 91 ORF sequences.

For phylogeographic inferences, based on the 144 sequences of the E gene, the analyses were performed using a standard continuous-time Markov chain (CTMC) and a Bayesian stochastic search variable selection (BSSVS) procedure that identifies the parsimonious descriptions of the diffusion process [42]. A constant-size coalescent process prior was assumed since Bayesian skyline plot model may have little influence on the phylogeographic inference [42]. A geographic location attributed was added to each taxon and the Brazilian DENV-2 isolates were categorized according to their geographic origin in Brazil (North, Northeast, South and Southeast regions). Additionally, a Bayes factor test was performed in order to identify well-supported rates to explain the diffusion process, using the platform SPREAD (spatial phylogenetic reconstruction of evolutionary dynamics) [43]. Rates yielding a Bayes factor higher than 3 were considered well supported diffusion rates [42]. The posterior distribution of ancestral location states was summarized using TreeAnotator v.1.6.1 [40] and the modal location state for each node was annotated on the maximum clade credibility tree using FigTree v.1.3.1 [41].

## Results

**Figure 1:**
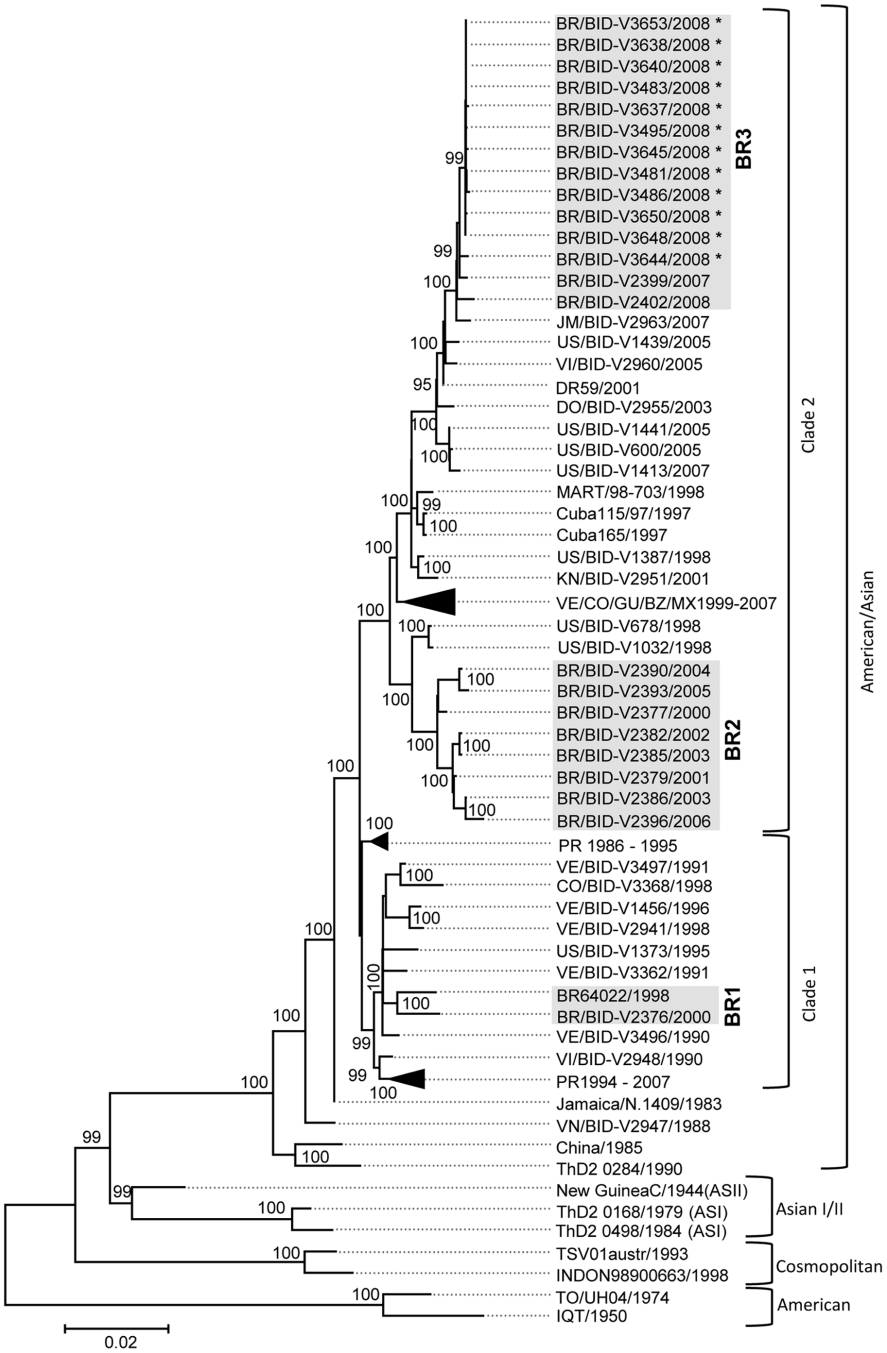
Phylogenetic analysis of DENV-2 based on the complete genome sequence. The evolutionary history was inferred using the Maximum Likelihood method, using the General Time Reversible model for nucleotide substitution with discrete Gamma distribution to model evolutionary rate differences among sites [4 categories (+*G*, parameter = 0.45)]. The tree with the highest log likelihood (−46330.60) is shown. The tree is drawn to scale, with branch lengths measured in the number of substitutions per site. The analysis involved 91 nucleotide sequences with a total of 10,167 positions in the final dataset. A total of 1000 bootstrap replicates were run and values ≥99 are represented as percentage in respective nodes. The Brazilian DENV-2 lineages are shown in grey. For clarity purposes some branches were collapsed. VE/CO/GU/BZ/MX 1999–2009 contains isolates from Venezuela (VE61095/2007, DENV-2/VE/BID-V2944/2005, -V2424/2004, -V2492/200, -V2216/2003, -V2262/2006), Colombia (DENV-2/CO/BID-V3370/2004, -V3369/1999, -V1603/2004), Guatemala (FDA-GUA09/2009), Belize (BZ/BID-V2952/2002) and Mexico (DENV-2MX/BID-V3661, -V3714 and –V3768); PR 1986–1995 contains isolates from Puerto Rico (DENV-2/US/BID-V1356/1993, -V855/1992, -V1182/1989, -V1175/1988, -V1183/1990, -V1171/1987, -V1164/1986, DENV-2/PR/17DN/1995 and DENV-2/PR/6780DN/1994 ) and PR 1994–2007 also contains isolates from Puerto Rico (DENV-2/US/BID-V37DN/1994, -V1424/1996, -V1427/1999, -V1398/1997, -V1038/1998, -V1367/1995, -V1463/2000, V1472/2001, -V593/2005 and –V1412/2007). Evolutionary analyses were conducted in MEGA5.0.

Phylogenetic analyses, based on the genome sequence, demonstrate that all Brazilian and other isolates from Central and South America and from the Caribbean grouped within American/Asian genotypes apart from strains from Southeast Asia (Figure 1). Brazilian strains are subdivided into three well-supported lineages (bootstrap values of 100%), which are placed in two different clades (Figure 1). Lineage BR1 groups two Brazilian strains isolated in 1998 and 2000 that also clusters strains from Venezuela, Colombia and Puerto Rico, isolated from 1990 to 1998. Lineage BR2 contains strains isolated from 2000 to 2006, in the Northern region of the country and these sequences are closely related to strains isolated in 1998 in Puerto Rico. Finally, the 12 DENV-2 strains sequenced here cluster together with two Brazilian strains from the North region, in a lineage, called BR3. These strains also cluster with a strain isolated in Jamaica in 2007. When a greater number of E sequences were included (total of 144 E sequences including 77 E sequences from Brazilian isolates), similar clustering patterns are observed in the Maximum likelihood tree (data not shown).

The 14th codon position in the NS3 gene, shows positive selection using all methods (with dN/dS >1 and p-values <0.05 for SLAC, FEL and IFEL, and posterior probability = 1.0 for FUBAR). When 77 predicted protein envelope sequences from Brazilian isolates were analyzed, seven amino acid (aa) substitutions are observed in domains I to III of this protein, differentiating the three lineages (Table 1).

**Table 1:** Amino acid differences in the envelope protein of Brazilian DENV-2 lineages.

Lineage	Amino acid position and localization in protein E						
	61(DII)	129(DII)	131(DII)	170(DI)	203(DII)	340(DIII)	380(DIII)
BR3	I	I	Q	T	D	T	V
BR2	V	V	Q	I	D	M	I^2^
BR1	I	V	L	I	E^1^	M	I

1 - except HQ012515.BR59382/RN/1997 and HQ012518.BR66985/RJ/2000 (K).

2 - except DENV-2/BR/BID-V2386/2003, DENV-2/BR/BID-V2396/2006, JF804028.BR/DB015/2006 (V).

Amino acid (aa) substitutions are observed in the deduced aa ORF sequences and some of them are unique to some lineages (Table S3). For BR1, 16 aa substitutions are observed in the deduced protein sequences [capsid (106I), membrane (134A), envelope (131L, 160E, 203E, 347A), NS2a (62V, 133V, 162L, 189T), NS3 (115L, 418R, 549R), NS5 (375R, 429S and 670L)] (Table S3); in BR2, six aa substitutions are observed [envelope (61V), NS2a (108V, 138K), NS3 (77V) NS5 (656T, 676N)] (Table S3) and finally, in BR3, 15 unique aa substitutions are observed [envelope (129I, 170T, 340T), NS1 (5I), NS2a (38L), NS3 (561K), NS4a (36V, 42T), NS4b (15F) and NS5 (5V,388E, 521E, 523G, 596K and 637A) (Table S3). One aa substitution is exclusive to 11 strains isolated in SJRP/SP in the NS5 sequence (position 874M) (Table S3). The aa substitution (63A) in the NS4a sequence is only observed in sequences obtained in SJRP/SP and another isolate from Rio de Janeiro (BR-BIDV2399/2007) (Table S3). Five aa substitutions [at M/prM (83T), E (129H), NS4a (64V), NS4b (17S) and at NS5 (891R)] are common to JM/BID-V2963/2007, US/BID-V1439/2005, VI/BID-V2960/2005 and all Brazilian isolates within BR3, with the exception of BR/BID-V2402/2008.

The estimate of average evolutionary divergence within the 12 strains from SJRP/SP is 0.074% ±0.012, based on the ORF sequence. The most divergent sequence is BR-BID-V3644/2008 when compared to the other 11 isolates. The nucleotide sequences from the whole ORF of four isolates BR/BID-V3653/2008, BR/BID-V3638/2008, BR/BID-V3640/2008 and BR/BID-V3483/2008 are identical to each other. The estimates of evolutionary divergence within each Brazilian lineage based on envelope sequences are BR1 = 1.05% ±0.11 (n = 32 sequences), BR2 = 0.68±0.14 (n = 8 sequences) and BR3 = 0.35±0.076 (n = 37 sequences). The estimates of evolutionary divergence among lineages BR1, BR2 and BR3 ranged from 2.77% ±0.40 to 3.10% ±0.39.

**Figure 2:**
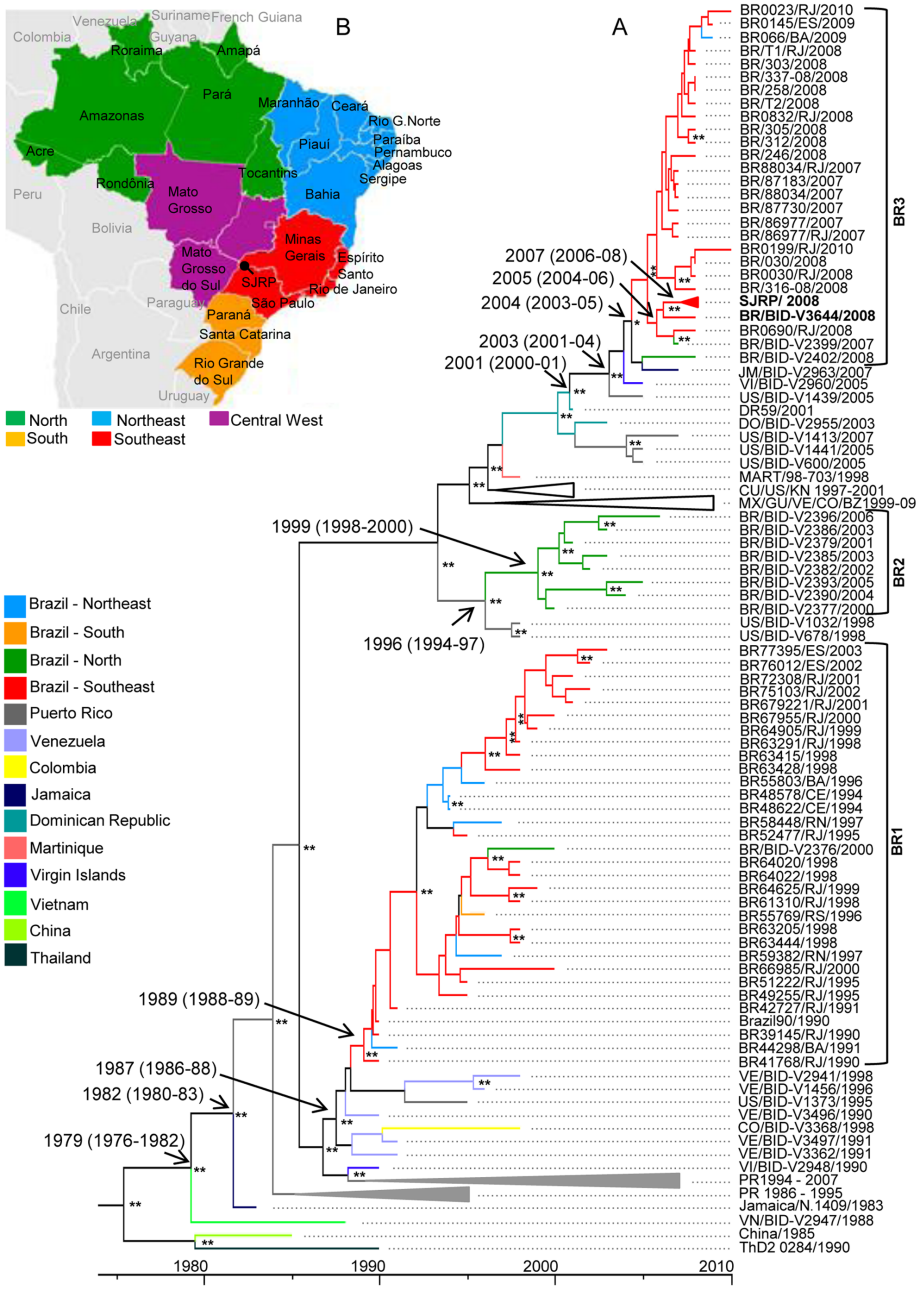
Bayesian coalescent and discrete phylogeography analyses of Brazilian DENV-2 based on envelope nucleotide sequence. Subtree of the maximum clade credibility tree was inferred using 144 DENV-2 envelope sequences (1,485 nt). The subtree containing isolates of the American/Asian genotype is displayed here. Time of the most recent common ancestor (MRCA) was estimated using the year of isolation as the calibration point, under the relaxed molecular clock, with the Tamura Nei Model, with discrete Gamma distribution and an estimated nucleotide substitution rate of 7.5^−4^. The posterior probabilities values ≥0.96 are represented by (*) and values ≥0.99 are represented by (**), inside the nodes. The years that the MRCA was estimated to exist are shown for some nodes with upper and lower intervals in parenthesis. The origin value of the reverse scale axis corresponds to year 2010. Using different colors, (legend shown on the left side), terminal branches were annotated based on geographic location of DENV-2 isolates. Internal nodes of the tree which presented modal state posterior probability ≥0.60 were also colored according to their most probable location states, inferred by discrete phylogeographical analysis. Brazilian lineages are delimited by square brackets. For clarity purposes some branches were collapsed. SJRP/2008 contains 11 isolates from São José do Rio Preto/São Paulo/Brazil (DENV-2/BR/BID-V3653/2008, -V3638/2008, -V3640/2008, -V3483/2008, -V3637/2008, -V3495/2008, -V3645/2008, -V3481/2008, -V3486/2008, -V3650/2008 and -V3648/2008); CU/US/KN 1997–2001 contains isolates from Cuba (Cuba115/97 and Cuba165/1997), Puerto Rico (US/BIC-V1387/1998) and Saint Kitts and Nevis (KN/BID-V2951/2001); VE/CO/GU/BZ/MX 1999–2009; PR 1986–1995 and PR 1994–2007 contain the same isolates as described in the Figure 1 caption. Analyses were performed using programs from BEAST package v.1.6.1, BEAUTi, Tracer v.1.5.0, TreeAnotator v.1.6.1 and FigTree v.1.3.1 (B) Map of Brazil showing the macro-regions and states. The black pin indicates the approximate location of São José do Rio Preto/São Paulo/Brazil.

The coalescent analyses were performed with a dataset containing 144 E sequences using the strict or relaxed (uncorrelated lognormal) molecular clock. Both analyses presented similar values (Table S4) and reached effective sample sizes >200 for all parameters and the results for the relaxed molecular clock are presented here. Based on the later analysis, the estimated mean overall substitution rate for the tree given in substitutions/site/year (ssy) ranged from 8.55×10^−4^ to 1.00×10^−3^ (95% HPD). Additionally, the MRCA of Latin America strains and the strain from Vietnam (VN/BID-V2947/1988) is estimated to have existed in 1979 [95% HPD = 1976–1982 (30.79±0.009 years before 2010, with 95% HPD = 28.15–33.83 yrs)] (Figure 2a), while the MRCA of all American/Asian genotype isolates probably existed in 1975 [95% HPD = 1971–1980 (34.57±0.01 years before 2010, with 95% HPD = 30.53–38.70 yrs)].

Similar clustering patterns of Brazilian DENV2 isolates were observed (Figure 2A) when compared to phylogenetic analyses (Figure 1), reinforcing the existence of three distinct Brazilian DENV-2 lineages. Lineage BR1 contains the oldest isolates and its MRCA is estimated to date at 1989 [95% HPD = 1988–1989 (20.94±0.002 years before 2010, with 95% HPD = 20.30–21.67 yrs)] These Brazilian strains comprise 32 isolates from 1990 to 2003, from different regions of the country including the South, North, and Northeast but predominantly from the Southeast (Figure 2 a and b). The MRCA of lineage BR1 is inferred to be from Southeast Brazil (Figure 2a) (posterior probability = 0.96) and this lineage is closely related to strains from Colombia, Venezuela and Puerto Rico sharing a MRCA with these later sequences dating at 1987 [95% HPD = 1986–1988 (22.39±0.004 years before 2010, with 95% HPD = 21.33–23.56 yrs)] (Figure 2a). When the MRCA of BR1 was estimated on the complete ORF sequence (based on sequences of only two isolates, BR/BID-V2376/2000 and BR64022/1998) it was calculated to have existed in 1995 [95% HPD = 1993–1997 (13.29±0.1 years before 2009, with 95% HPD = 11.50–15.53 yrs)] (data not shown). The MRCA for these two Brazilian DENV-2 isolates (BR/BID-V2376/2000 and BR64022/1998) was also calculated based on the E sequence and it is also estimated to have existed in 1995 [95% HPD = 1992–1997 (14.53±0.04 years before 2010, with 95% HPD = 12.76–17.47 yrs)] (data not shown).

Eight Brazilian isolates are clustered within lineage BR2, including strains obtained from 2001 to 2006 in the Northern region (Figure 2a and b). The MRCA of BR2 is estimated to be from Northern Brazil (Figure 2a) (posterior probability = 0.96), dating at 1999 [95% HPD = 1998–2000 (11.00±0.003 years before 2010, with 95% HPD = 10.17–11.92 yrs)] (Figure 2a). The MRCA of BR2 estimated on the complete ORF sequence is also calculated to have existed in 1999 [95% HPD = 1998–2000 (10.06±0.01 years before 2009, with 95% HPD = 9.31–10.88 yrs)] (data not shown). This lineage is closely related to two strains from Puerto Rico, sharing a MRCA estimated to date at 1996 [95% HPD = 1994–1997 (13.97±0.004 years before 2010, with 95% HPD = 10.19–11.93 yrs)] and inferred to be from Puerto Rico (Figure 2a) (posterior probability = 0.60).

Lineage BR3 is the youngest lineage grouping 37 strains isolated from 2007 to 2010 mainly in the Southeast and also North and Northeast regions of the country (Figure 2a and b). These Brazilian isolates are closely related to a strain from Jamaica sharing the MRCA with this later strain (JM/BID-V2963/2007), which probably dates at 2004 [95% HPD = 2003–2005 (5.74±0.004 before 2010, with 95% HPD = 4.68–6.97 yrs) (Figure 2a). The MRCA of BR3 estimated on the complete ORF sequence was calculated to have existed in 2005 [95% HPD = 2004–2006 (3.41±0.01 years before 2009, with 95% HPD = 2.57–4.24 yrs)] (data not shown). Isolates from BR3 and Jamaica (JM/BID-V2963/2007) are also closely related to strains isolated in Puerto Rico (US/BID-V1439/2005) and the Virgin Islands (VI/BID-2960/2005) with an estimated date for the MRCA of 2003 [95% HPD = 2001–2004 (6.99±0.02 years before 2010, with 95% HPD = 5.57–8.77 yrs) (Figure 2a). These isolates altogether plus another one from the Dominican Republic (DR59/2001) share the MRCA inferred to be from the Dominican Republic (Figure 2a) (posterior probability = 0.87) and it probably dates at 2001 [95% HPD = 2000–2001 (9.0±0.001 years before 2010, with 95% HPD = 9.0–9.59 yrs) (Figure 2a).

All strains from SJRP/SP, except BR/BID-V3644/2008, have a common ancestor estimated to date at 2007 [95% HPD = 2006–2008 (2.87±0.003 years before 2010, with 95% HPD = 2.15–3.53 yrs)] (Figure 2a). All strains from SJRP/SP and two strains from the North and Southeast regions (BR/BID-V2399/2007 and BR0690/RJ/2008) have a MRCA probably from Southeast Brazil (Figure 2a and b) (posterior probability = 1.0) dating at 2005 [95% HPD = 2004–2006 (4.37±0.003 years before 2010, with 95% HPD = 3.39–5.31 yrs)] (Figure 2a).

Well-supported diffusion rates with Bayes Factor (BF) higher than 3 (data not shown) are observed supporting different migration pathways among countries from Central America (Mexico, Belize and Guatemala), among the Caribbean island countries (Dominican Republic, Virgin Islands, Puerto Rico, Saint Kitts and Nevis, Jamaica, Cuba and Martinique) and between Venezuela and Colombia. Well-supported rates supporting different migration pathways within Brazil are also observed, such as between the Northeast and Southeast (BF>3000), between the North and Southeast (BF = 27) and between the Northeast and South (BF = 12). Additionally, it is also noted that there is a migration pathway with a well-supported rate (BF = 4) between Northern Brazil and the Virgin Islands.

## Discussion

Our study demonstrates that the isolates from SJRP/SP all group within the American/Asian genotype together with isolates from South and Central America and the Caribbean, as previously demonstrated for other Brazilian isolates [21]–[22]; [44]. This genotype was most probably introduced into the Americas from Vietnam via Cuba, approximately 30 years ago [8]. Indeed, our estimates for the MRCA for the Latin America cluster plus one Vietnam strain as well as the MRCA for the American/Asian genotypes are in agreement with previous findings [8]; [45]–[46], thereby supporting our current results.

Brazilian isolates were subdivided into three well-defined lineages and 37 unique aa substitutions were mapped to the predicted protein sequence from the entire ORF that characterized and differentiated these lineages. In particular some aa differences were observed in the deduced envelope protein sequences unique among BR1, BR2 and/or BR3: three aa differences were observed between BR1 and BR2, five aa differences were observed between BR2 and BR3 and finally, six aa differences were observed between BR1 and BR3. Although, none of those aa differences in the envelope sequence were associated with positively selected codons in this study, they were located in the domains I, II and III [47] which are known to be involved in the production of neutralizing antibodies against DENV *in vivo* or *in vitro* [48]–[50]. Moreover, residue 131 is located within a pH-dependent hinge region at the interface between domains I and II of the protein, and mutations in this region may affect the pH threshold fusion of envelope protein to membrane of the target cell [47]. It is possible that the introduction and establishment of different DENV-2 lineages in Brazil could have been favored by aa differences that were involved in the immunologic escape from antibodies produced against previous DENV-2 lineages, by co-circulating DENV-1 and DENV-3, [14], [16] or even due to stochastic events [6].

The oldest lineage, BR1, is estimated to have been introduced between 1988 and 1989. This lineage contains strains isolated from 1990 to 2003, from a wider geographic region and represents a longer time period (14 years) than the other lineages. BR1 also has the highest values with regard to genetic diversity followed by BR2 and then BR3. The isolates responsible for the epidemics in 1990 and 1998 were clustered within this lineage. In 1990, an epidemic outbreak took place in Rio de Janeiro one or two years after the estimated date of introduction of the American/Asian genotype into the country. During this outbreak, severe illness and fatal cases were observed and the virus spread throughout the country [12]; [19]–[20]. During the epidemic in 1998, more than 528,000 dengue cases were registered in Brazil [51] with the occurrence of more severe clinical presentations when compared to DENV-1 outbreaks [14]. Within BR1, there is no clear division between the strains from the 1990 and 1998 epidemics, however the 1990–1991 strains are basal to later strains and suggest local evolution up to 2003. This was also observed in other studies, suggesting that viruses circulating in the 1990 and 1998 epidemics belong to the same lineage, most likely introduced in Rio de Janeiro (Southeast regions) [21]–[22]; [44], from neighboring South America countries such as Venezuela and Paraguay [22]. In fact, the MRCA of lineage BR1 was estimated to be from Southeast Brazil supporting the idea that this lineage was probably introduced into that region and then disseminated to other parts of the country, which is also in accordance with its geographic occurrence in four different regions and with the inferred migration pathways of DENV-2 within the country. Additionally, a close relationship between lineage BR1 and strains from South American countries (Venezuela and Colombia) as well as Puerto Rico was observed. Although it was not possible to confirm the epidemiological linkage between Brazil, Venezuela, Colombia or Puerto Rico, this region appears to be a possible ancestral location of strains circulating in South America countries.

BR2 is the second oldest lineage, estimated to have been introduced in Northern Brazil probably from Puerto Rico between 1998 and 2000 and it was not associated with any major reported epidemics. This lineage was restricted to Northern Brazil with only eight sampled strains from 2000 to 2006. Previous studies have demonstrated that serotype and/or genotype co-circulation in a particular region and serotype displacement may result in complex patterns of competition, affecting population diversity and lineage turnover [52]–[53].The introduction of DENV-3 in the Southeast region, likely in 1999 and then in the North region of the country in 2001 [16] could be responsible for the restricted circulation of BR2 at that time. Likewise, this later scenario is also in agreement with the conditions observed during the last DENV-2 epidemics in Brazil (2007/08 and 2010) which may explain the emergence of lineage BR3: a shift from the DENV-3 to DENV-2 prevalence in Southern Brazil and the introduction (at least in 2005) and emergence (in 2007) of a genetically distinct lineage [22].

BR3 is the most recent lineage to emerge in Brazil, grouping isolates circulating from 2007 through at least 2010, primarily from the Southeast region (particularly São Paulo and Rio de Janeiro states). Some studies suggested that lineage BR3 has been introduced into Brazil from the Caribbean [22], [44]. Our results demonstrate that isolates JM/BID-V2963/2007 (from Jamaica), VI/BID-V2960 (from Virgin Islands) and US/BID-V1439/2005 (from Puerto Rico) are the closest strains to lineage BR3. Although a possible migration pathway for DENV-2 between Northern Brazil and the Virgin Islands was observed it was not possible to identify (with statistic support) if any of these countries is the ancestral location of the MRCA of BR3. On the other hand, all these strains together in addition to a strain from the Dominican Republic probably have a MRCA from this later country, dating at 2001 giving support to the idea that lineage BR3 originated from the Caribbean.

Our results are in agreement with previous estimates that indicated that this lineage was already circulating in the country before its first detection in 2007 [22]. Similar patterns of introduction/detection were also observed for DENV-2 lineages BR1 and BR2 in this study, for DENV-1 [13] and DENV-3 [16] in Brazil and for DENV serotypes (1 to 4) in the Americas [53]. This delay between the introduction of DENV in a region and its detection could be explained by the fact that viruses can remain undetected until the number of infections and/or disease incidence reaches a threshold of detection that is high due to poor surveillance in most countries in the Americas [46].

Coalescent analysis performed on envelope sequences demonstrated that within lineage BR3, there is a subdivision with BR/BID/V-2402/2008 and JM/BID-V2963/2007 being more related to each other and sharing a common ancestor at 2004/05 (5.38 yrs before 2010) while all other isolates within BR3 share a common ancestor at 2005 (4.82 yrs before 2010), probably from Southeast Brazil. Based on these findings and the five aa substitutions that are common to JM/BID-V2963/2007, US/BID-V1439/2005, VI/BID-V2960/2005 and all Brazilian isolates within BR3, excepting BR/BID-V2402/2008, it is probable that strains circulating in the Southeast region did not directly originate from BR/BID/V-2402/2008, detected in the Northern region. This is also supported by the observation of two unique aa substitutions [NS4a (63A) and at NS5 (412V)] observed in all sequences from BR3 with the exception of BR/BID-V2402/2008. One can speculate that this lineage might have been introduced into Brazil by two parallel events or that after the introduction, the virus disseminated through the country, which is also supported by the different inferred migration pathways of DENV-2 within the country, and it has been locally evolving giving rise to different strains, in the North and Southeast regions. We observed that isolates from SJRP/SP formed a homogeneous group with low genetic diversity and the most divergent strain was BR-BID-V3644/2008. Isolates from SJRP/SP were close to isolates from Rio de Janeiro, having been introduced into SJRP/SP, probably from Southeast Brazil, at least by 2007, prior to detection in the 2008 outbreak in SJRP/SP.

DENV-2 circulation in Brazil is characterized by introduction of three different lineages over time, most probably from the Caribbean. The Caribbean region and other South American countries have been proposed to be the main sources of DENV introduction into Brazil [22], [46]. The close geographic proximity of the Northern region to Caribbean countries favors the introduction of DENV from the Caribbean into that region [16], with similar situations being valid for different regions of Brazil and their neighboring countries [13], [21], [22], [44], [54]. The Southeast region, and especially the state of Rio de Janeiro has been considered to be the most important point of DENV introduction into the country, since this was the state where the first cases of DENV-1, DENV-2 and DENV-3 were reported [14]. Southeast Brazil concentrates the largest and the most densely inhabited cities/states, including São Paulo and Rio de Janeiro. These states contain the most important airports in the country and they are also well connected to other regions via land transport systems [16]. It’s likely that lineages BR1 and BR3 were first introduced to and/or disseminated through the Southeast region, which might be one of the factors contributing to the apparent greater distribution of these lineages throughout the country. Dengue outbreaks that took place in Brazil in 2007/2008 and 2010, related to lineage BR3, were more severe in terms of overall number of cases, and number of severe cases as well as number of deaths, than the previous DENV-2 epidemics in 1990 and 1998, which were related to lineage BR1. It is possible, given the severity of the more recent epidemics, that in addition to the co-circulation of DENV-1 and DENV-3 during these years, the genetic characteristics of DENV-2 BR3 could have contributed to different biological characteristics, which in turn might have played a role in the displacement of other serotypes or lineages circulating in some regions of the country.

In Brazil, the co-circulation of different lineages has been demonstrated for DENV-1 and DENV-3 [13], [16] and co-circulation of different genotypes has been demonstrated for DENV-4 [17]. However, in case of DENV-2, the introduction of new lineages was associated with the displacement of the old lineage and the emergence of a new one. Apparently, the co-circulation of DENV-2 BR1 and BR2 occurred for only a short period of time. This pattern is dissimilar to the DENV-2 dynamics observed in Puerto Rico, where different lineages co-circulate and one endemic strain seems to be refractory to influences from frequent foreign DENV-2 strains [55].

Phylogenetic analyses have demonstrated a constant clade or lineage turnover process in DENV dynamics [52], [56]. In addition to the introduction and co-circulation of different serotypes, genotypes or lineages, that may have different spatiotemporal distributions, the lineage replacements might be associated with epidemic outbreaks. Indeed, major epidemic outbreaks took place a few years after the introduction of BR1 and BR3 into the country. The high migration rates of DENV-2 between the Caribbean, Central and South America and even throughout Brazil, in addition to the observed delay between the introduction of a new virus (serotype/genotype/lineage) in one area and its detection, reinforces the need for surveillance programs in these regions in order to detect and trace the evolution of these viruses as soon as possible. This could help to establish measures to prevent significant outbreaks and to lower the number of severe dengue cases and deaths. Moreover this genetic diversity also should be taken into account in dengue control programs, since the valuation of vaccine efficacy should consider different epidemiological situations added to a range of dengue viruses with distinct virulence and viral fitness circulating in both epidemic and endemic context [57]. Finally, our data indicate the existence of three well-supported DENV-2 lineages in Brazil and we also dated, for the first time, the introduction of the American/Asian genotype into Brazil. The Brazilian DENV-2 lineages differed in genetic diversity, year of introduction, origin and geographic distribution in Brazil, which are important features when considering evolution, dynamics and control of dengue and dengue viruses.

## Supporting Information

Table S1 **PCR and sequencing primer sequences.**
(DOC)

Table S2 **GenBank accession numbers of Dengue virus type 2 sequences used in this study regarding selection, phylogenetic, phylogeographic and coalescent analyses.**
(DOC)

Table S3 **Amino acids substitutions observed in the whole deduced sequence of DENV-2, characterizing different lineages of Dengue virus 2 from Brazil and some strains from Latin America.**
(DOC)

Table S4 **Estimate of Most Recent Common Ancestor (MRCA) of Brazilian DENV-2 lineages, based on envelope gene sequence.**
(DOC)
