# Supplementary material for: Circulation of Different Lineages of Dengue Virus 2, Genotype American/Asian in Brazil: Dynamics and Molecular and Phylogenetic Characterization
Source: PLoS One. 2013 Mar 22;8(3):e59422. doi: 10.1371/journal.pone.0059422 (PMC3606110; doi:10.1371/journal.pone.0059422)
Supplement: Table S1 — PCR and sequencing primer sequences. (DOC) [file pone.0059422.s001.doc]

**Table S1 - PCR and sequencing primer sequences**

Forward PCR primers

| **1st Round Fragment Size** | **Primer_Forward** | **Seq** | **gene applified** |
| --- | --- | --- | --- |
| 1748 | broad_D2_F_11 | CTACGTGGACCGACAAAGACAG | C,PreM, M,E |
| 2074 | broad_D2_F_11 | CTACGTGGACCGACAAAGACAG | C,PreM, M,E |
| 1976 | broad_D2_F_1093 | CCTGCCACCCTAAGGAAGTACT | E, NS1 |
| 1877 | broad_D2_F_1117 | atagaggcaaagctgaccaac | E, NS1 |
| 2063 | broad_D2_F_2951 | tattctgcgactcaaactcatg | NS1,NS2A,NS2B |
| 2106 | broad_D2_F_2951 | tattctgcgactcaaactcatg | NS1,NS2A,NS2B,NS3 |
| 1702 | broad_D2_F_3549 | GACCCGAGTAGGAACGAAACAT | NS2A,NS2B,NS3 |
| 1992 | broad_D2_F_3760 | aagttgacctccaaggaattg | NS2A,NS2B,NS3 |
| 1718 | broad_D2_F_5209 | gaaatggaggaagccctaaga | NS3, NS4A |
| 2060 | broad_D2_F_5533 | GAAATCCCTGAACGTTCRTGGA | NS3, NS4A,NS4B |
| 1962 | broad_D2_F_6886 | cagcaacccgagagcaacatc | NS4A,NS4B,NS5 |
| 1997 | broad_D2_F_7571 | GAACTGGCAACATAGGAGAGA | NS4B,NS5 |
| 2040 | broad_D2_F_8033 | GGACGAACACTCAGAGTCCTTA | NS5 |
| 1740 | broad_D2_F_8548 | CTGCTGACAAAACCTTGGGA | NS5 |
| 926 | broad_D2_F_10246 | gaggaagaggcaggagtcctg | NS5,C |
| 757 | broad_D2_F_10246 | gaggaagaggcaggagtcctg | NS5,C |

Reverse PCR primers

| **1st Round Fragment Size** | **Rvs Primer Name** | **Seq** | **gene applified** |
| --- | --- | --- | --- |
| 1748 | broad_D2_R_1738 | tgatgacatctggatttctgt | C,PreM, M,E |
| 2074 | broad_D2_R_2068 | CGGCTCTACTCCTATGATGAT | C,PreM, M,E |
| 1976 | broad_D2_R_3048 | GAGGCTTTCTCTATCTTCCAT | E, NS1 |
| 1877 | broad_D2_R_2974 | gttgtcttttatggccgctga | E, NS1 |
| 2063 | broad_D2_R_4993 | agcactcacatatgctccact | NS1,NS2A,NS2B |
| 2106 | broad_D2_R_5036 | CGATCTCTGGGTTGTCTTCAA | NS1,NS2A,NS2B,NS3 |
| 1702 | broad_D2_R_5219 | ttggtatcttattggaagtcc | NS2A,NS2B,NS3 |
| 1992 | broad_D2_R_5717 | AATGTCAGTTGTAACCACGAAATC | NS2A,NS2B,NS3 |
| 1718 | broad_D2_R_6907 | gggacgtagatctatgtccag | NS3, NS4A |
| 2060 | broad_D2_R_7572 | GTCTCTCCTATGTTGCCAGTT | NS3, NS4A,NS4B |
| 1962 | broad_D2_R_8827 | aacagcctcacgtgccgactt | NS4A,NS4B,NS5 |
| 1997 | broad_D2_R_9536 | aacacaatcatctccactgat | NS4B,NS5 |
| 2040 | broad_D2_R_10050 | GTATGGGATTTCCTCCCATGATT | NS5 |
| 1740 | broad_D2_R_10264 | tgttagttttgccttctaccacag | NS5 |
| 926 | broad_D2_R_449 | GTGTGGTTCTCCGTTACGTGTGG | NS5,C |
| 757 | broad_D2_R_280 | tctcttcagtatccctgctgt | NS5,C |
