# Supplementary material for: Circulation of Different Lineages of Dengue Virus 2, Genotype American/Asian in Brazil: Dynamics and Molecular and Phylogenetic Characterization
Source: PLoS One. 2013 Mar 22;8(3):e59422. doi: 10.1371/journal.pone.0059422 (PMC3606110; doi:10.1371/journal.pone.0059422)
Supplement: Table S2 — GenBank accession numbers of Dengue virus type 2 sequences used in this study regarding selection, phylogenetic, phylogeographic and coalescent analyses. (DOC) [file pone.0059422.s002.doc]

**Table S2. GenBank accession numbers of Dengue virus type 2 sequences used in this study regarding selection, phylogenetic, phylogeographic and coalescent analyse**s.

| Sequence /analysis | GenBank accession numbers |
| --- | --- |
| 91 complete genome sequences / phylogenetic / coalescent analyses | AB122022, AB189122, AF038403, AF119661, AF208496, AF489932, AY037116, AY702036, AY702038, DQ181801, DQ181804, DQ181805, EU056811, EU482545, EU482550, EU482568, EU482575, EU482578, EU482585, EU482586, EU482594, EU482724, EU482731, EU482735, EU569705, EU569706, EU569708, EU569716, EU596490, EU596491, EU677141, EU677144, EU687212, EU687216, EU687217, EU687220, EU687224, EU687232, FJ024477, FJ639783, FJ639822, FJ850072, FJ850074, FJ850076, FJ850078, FJ850082, FJ850085, FJ850088, FJ850091, FJ850112, FJ898450, FJ898451, FJ898453, FJ898460, FJ898461, FJ898465, FJ898466, FJ898467, GQ199890, GQ199892, GQ398283, GQ398301, GQ398311, GQ868516, GQ868540, GQ868541, GQ868549, GQ868550, GQ868551, GQ868552, GQ868553, GQ868554, GQ868595, GQ868640, GU131864, GU131879, GU131880, GU131881, GU131882, GU131883, GU131884, GU131885, GU131955, GU131959, HM181971, HM582117, HQ332190 HQ999999, JN819418, JN819419, M20558, |
| 144 envelope sequences /  phylogenetic/ coalescent/ phylogeographic analyses | AB122022, AB189122, AF038403, AF119661, AF208496, AF489932, AY037116, AY577430, AY702036, AY702038, DQ181801, DQ181804, DQ181805, EU056811, EU482545, EU482550, EU482568, EU482575, EU482578, EU482585, EU482586, EU482594, EU482724, EU482731, EU482735, EU569705, EU569706, EU569708, EU569716, EU596490, EU596491, EU677141, EU677144, EU687212, EU687216, EU687217, EU687220, EU687224, EU687232, FJ024477, FJ639783, FJ639822, FJ850072, FJ850074, FJ850076, FJ850078, FJ850082, FJ850085, FJ850088, FJ850091, FJ850112, FJ898450, FJ898451, FJ898453, FJ898460, FJ898461, FJ898465, FJ898466, FJ898467, GQ199890, GQ199892, GQ368158, GQ368159, GQ368160, GQ368161, GQ368162, GQ368163, GQ368164, GQ368165, GQ368166, GQ368167, GQ368168, GQ368169, GQ368170, GQ368171, GQ368172, GQ368173, GQ368174, GQ368175, GQ368176, GQ398283, GQ398301, GQ398311, GQ868516, GQ868540, GQ868541, GQ868549, GQ868550, GQ868551, GQ868552, GQ868553, GQ868554, GQ868595, GQ868640, GU131864, GU131879, GU131880, GU131881, GU131882, GU131883, GU131884, GU131885, GU131955, GU131959, HM181971, HM582117, HQ012508, HQ012509, HQ012510, HQ012511, HQ012512, HQ012513, HQ012514, HQ012515, HQ012516, HQ012517, HQ012518, HQ012519, HQ012520, HQ012521, HQ012522, HQ012523, HQ012524, HQ012525, HQ012526, HQ012527, HQ012528, HQ012529, HQ012530, HQ012531, HQ012532, HQ012533, HQ012534, HQ012535, HQ012536, HQ012537, HQ012538, HQ026763, HQ332190, HQ999999, JN819418, JN819419, L10041, M20558, |
| 100 genome sequences/  selection analyses | AB122020, AB122022, AF208496, AF489932, AY702036, AY702038, AY702039, EU482545, EU482546, EU482550, EU482560, EU482568, EU482570, EU482572, EU482575, EU482578, EU482580, EU482585, EU482586, EU482594, EU482607, EU482724, EU482731, EU482735, EU529701, EU529701, EU569705, EU569706, EU569708, EU569716, EU596490, EU596491, EU677141, EU677142, EU677144, EU677145, EU687212, EU687216, EU687217, EU687220, EU687224, EU687232, EU687240, FJ024477, FJ639822, FJ850072, FJ850074, FJ850076, FJ850078, FJ850082, FJ850085, FJ850088, FJ850091, FJ850108, FJ850112, FJ850112, FJ898450, FJ898451, FJ898453, FJ898460, FJ898461, FJ898465, FJ898466, FJ898467, GQ199890, GQ199892, GQ199893, GQ199894, GQ398283, GQ398284, GQ398301, GQ398311, GQ868516, GQ868540, GQ868541, GQ868549, GQ868551, GQ868552, GQ868554, GQ868595, GQ868603, GQ868640, GU131864, GU131880, GU131881, GU131882, GU131883, GU131884, GU131947, GU131955, GU131959, HM181971, HQ332190, HQ999999, JN819408, JN819418, JN819418, JN819419, JN819422, M20558. |
