# Supplementary material for: Circulation of Different Lineages of Dengue Virus 2, Genotype American/Asian in Brazil: Dynamics and Molecular and Phylogenetic Characterization
Source: PLoS One. 2013 Mar 22;8(3):e59422. doi: 10.1371/journal.pone.0059422 (PMC3606110; doi:10.1371/journal.pone.0059422)
Supplement: Table S3 — Amino acids substitutions observed in the whole deduced sequence of DENV-2, characterizing different lineages of Dengue virus 2 from Brazil and some strains from Latin America. (DOC) [file pone.0059422.s003.doc]

**Table S3. Amino acids substitutions observed in the whole deduced sequence of DENV-2, characterizing different lineages of Dengue virus 2 from Brazil**

| L | Strains | Protein and amino acid position | | | | | | | | | | |
| --- | --- | --- | --- | --- | --- | --- | --- | --- | --- | --- | --- | --- |
|  |  | capsid | | | | | M and prM | | | | | |
|  |  | 25 | 102 | 104 | 106 | 112 | 29 | 39 | 83 | 134 | 135 | 151 |
| BR3 | **BR/BID-V2402/2008** | T | A | V | V | A | D | I | I | T | I | R |
| BR3 | **BR/BID-V2399/2007** | . | . | . | . | . | . | M | T | . | . | . |
| BR3 | ***BR/BID-V3644/2008*** | . | . | . | . | . | . | . | T | . | . | . |
| BR3 | ***BR/BID-V3648/2008*** | . | . | . | . | . | . | . | T | . | . | . |
| BR3 | ***BR/BID-V3650/2008*** | . | . | . | . | . | . | . | T | . | . | . |
| BR3 | ***BR/BID-V3653/2008**** | . | . | . | . | . | . | . | T | . | . | . |
| BR3 | ***BR/BID-V3481/2008*** | . | . | . | . | . | . | . | T | . | . | . |
| BR3 | ***BR/BID-V3495/2008*** | . | . | . | . | . | . | . | T | . | . | . |
| BR3 | ***BR/BID-V3645/2008*** | . | . | . | . | . | . | . | T | . | . | . |
| BR3 | ***BR/BID-V3486/2008*** | . | . | . | . | . | . | . | T | . | . | . |
| BR3 | ***BR/BID-V3637/2008*** | . | . | . | . | . | . | . | T | . | . | . |
|  | JM/BID-V2963/2007 | . | . | . | . | . | . | . | T | . | . | . |
|  | US/BID-V1439/2005 | . | . | . | . | . | . | . | T | . | . | . |
|  | VI/BID-V2960/2005 | . | . | . | . | . | . | . | T | . | . | . |
| BR2 | **BR/BID-V2377/2000** | . | . | . | . | . | . | . | T | . | . | . |
| BR2 | **BR/BID-V2390/2004** | . | V | . | . | . | N | . | T | . | . | . |
| BR2 | **BR/BID-V2393/2005** | . | V | . | . | . | N | . | T | . | . | . |
| BR2 | **BR/BID-V2382/2002** | . | . | A | . | V | . | . | T | . | M | . |
| BR2 | **BR/BID-V2385/2003** | . | . | A | . | V | . | . | T | . | M | . |
| BR2 | **BR/BID-V2379/2001** | S | . | . | . | V | . | . | T | . | M | . |
| BR2 | **BR/BID-V2386/2003** | . | . | . | . | V | . | . | T | . | M | . |
| BR2 | **BR/BID-V2396/2006** | . | . | . | . | V | . | . | T | . | M | . |
|  | US/BID-V678/1998 | . | . | . | . | . | . | . | T | . | . | . |
|  | US/BID-V1032/1998 | . | . | . | . | . | . | . | T | . | . | . |
| BR1 | **BR64022** | . | . | . | I | . | . | . | T | A | . | . |
| BR1 | **BR/BID-V2376/2000** | . | . | . | I | . | . | M | T | A | . | . |
|  | VE/BID-V1456/1996 | . | . | . | I | . | . | . | T | A | . | K |
|  | VE/BID-V2941/1998 | . | . | . | I | . | . | . | T | A | . | K |

Table S3. Amino acids substitutions observed in the whole deduced sequence of DENV-2, characterizing different lineages of Dengue virus 2 from Brazil (continued).

| L | Strains | Protein and amino acid position | | | | | | | | | | | | | | | | | |
| --- | --- | --- | --- | --- | --- | --- | --- | --- | --- | --- | --- | --- | --- | --- | --- | --- | --- | --- | --- |
|  |  | Envelope | | | | | | | | | | | | | | | | | |
|  |  | 61 | 129 | 131 | 140 | 149 | 160 | 162 | 170 | 203 | 331 | 340 | 347 | 359 | 365 | 380 | 432 | 447 | 449 |
| BR3 | **BR/BID-V2402/2008** | I | I | Q | V | R | K | I | T | D | S | T | V | T | V | V | I | A | S |
| BR3 | **BR/BID-V2399/2007** | . | . | . | . | H | . | . | . | . | . | . | . | . | . | . | . | . | . |
| BR3 | ***BR/BID-V3644/2008*** | . | . | . | . | H | . | . | . | . | . | . | . | . | . | . | . | . | . |
| BR3 | ***BR/BID-V3648/2008*** | . | . | . | . | H | . | . | . | . | . | . | . | . | . | . | . | . | . |
| BR3 | ***BR/BID-V3650/2008*** | . | . | . | . | H | . | . | . | . | . | . | . | . | . | . | . | . | . |
| BR3 | ***BR/BID-V3653/2008**** | . | . | . | . | H | . | . | . | . | . | . | . | . | . | . | . | . | . |
| BR3 | ***BR/BID-V3481/2008*** | . | . | . | . | H | . | . | . | . | . | . | . | . | . | . | . | . | . |
| BR3 | ***BR/BID-V3495/2008*** | . | . | . | . | H | . | . | . | . | . | . | . | . | . | . | . | . | . |
| BR3 | ***BR/BID-V3645/2008*** | . | . | . | . | H | . | . | . | . | . | . | . | . | . | . | . | . | . |
| BR3 | ***BR/BID-V3486/2008*** | . | . | . | . | H | . | . | . | . | . | . | . | . | . | . | . | . | . |
| BR3 | ***BR/BID-V3637/2008*** | . | . | . | . | H | . | . | . | . | . | . | . | . | . | . | . | . | . |
|  | JM/BID-V2963/2007 | . | . | . | . | H | . | . | . | . | A | . | . | . | . | . | . | . | . |
|  | US/BID-V1439/2005 | . | . | . | . | H | . | . | . | . | . | . | . | . | . | . | . | . | . |
|  | VI/BID-V2960/2005 | . | . | . | . | H | . | . | . | . | . | . | . | . | . | . | . | . | . |
| BR2 | **BR/BID-V2377/2000** | V | V | . | . | H | . | . | I | . | . | M | . | . | . | I | . | . | . |
| BR2 | **BR/BID-V2390/2004** | V | V | . | . | H | . | . | I | . | . | M | . | . | . | I | . | . | . |
| BR2 | **BR/BID-V2393/2005** | V | V | . | . | H | . | . | I | . | . | M | . | A | . | I | . | . | . |
| BR2 | **BR/BID-V2382/2002** | V | V | . | . | H | . | . | I | . | . | M | . | . | . | I | . | . | . |
| BR2 | **BR/BID-V2385/2003** | V | V | . | . | H | . | . | I | . | . | M | . | . | . | I | . | . | . |
| BR2 | **BR/BID-V2379/2001** | V | V | . | . | H | . | . | I | . | . | M | . | . | . | I | . | . | . |
| BR2 | **BR/BID-V2386/2003** | V | V | . | . | H | . | . | I | . | . | M | . | . | . | . | . | . | N |
| BR2 | **BR/BID-V2396/2006** | V | V | . | . | H | . | V | I | . | . | M | . | . | . | . | . | . | N |
|  | US/BID-V678/1998 | . | . | . | M | H | . | . | I | . | . | M | . | . | . | I | V | . | . |
|  | US/BID-V1032/1998 | . | . | . | M | H | . | . | I | . | . | M | . | . | . | I | V | . | . |
| BR1 | **BR64022** | . | V | L | . | H | E | . | I | E | . | M | A | . | . | I | . | V | . |
| BR1 | **BR/BID-V2376/2000** | . | V | L | . | H | E | . | I | E | . | M | A | . | . | I | . | . | . |
|  | VE/BID-V1456/1996 | . | V | L | . | H | . | . | I | E | . | M | . | . | I | I | . | . | . |
|  | VE/BID-V2941/1998 | . | V | L | . | H | . | . | I | E | . | M | . | . | . | I | . | . | . |

Table S3. Amino acids substitutions observed in the whole deduced sequence of DENV-2, characterizing different lineages of Dengue virus 2 from Brazil (continued).

| L | Strains | Protein and amino acid position | | | | | | | | | | | | | | |
| --- | --- | --- | --- | --- | --- | --- | --- | --- | --- | --- | --- | --- | --- | --- | --- | --- |
|  |  | NS1 | | | | | | | | | | | NS2a | | | |
|  |  | 5 | 121 | 125 | 146 | 164 | 188 | 190 | 212 | 261 | 275 | 278 | 12 | 32 | 33 | 38 |
| BR3 | **BR/BID-V2402/2008** | I | A | S | T | T | I | D | M | H | M | D | V | A | I | L |
| BR3 | **BR/BID-V2399/2007** | . | . | . | . | . | . | . | . | . | . | . | . | . | . | . |
| BR3 | ***BR/BID-V3644/2008*** | . | . | . | . | . | . | N | . | . | . | . | . | . | . | . |
| BR3 | ***BR/BID-V3648/2008*** | . | . | . | . | . | . | . | . | . | . | . | . | . | . | . |
| BR3 | ***BR/BID-V3650/2008*** | . | . | . | . | . | . | . | . | . | . | . | . | . | . | . |
| BR3 | ***BR/BID-V3653/2008**** | . | . | . | . | . | . | . | . | . | . | . | . | . | . | . |
| BR3 | ***BR/BID-V3481/2008*** | . | . | . | . | . | . | . | . | . | . | . | . | . | . | . |
| BR3 | ***BR/BID-V3495/2008*** | . | . | . | . | . | . | . | . | . | . | . | . | . | . | . |
| BR3 | ***BR/BID-V3645/2008*** | . | . | . | . | . | . | . | . | . | . | . | . | . | . | . |
| BR3 | ***BR/BID-V3486/2008*** | . | V | . | . | . | . | . | . | . | . | . | . | . | . | . |
| BR3 | ***BR/BID-V3637/2008*** | . | . | . | . | . | . | . | . | . | I | . | . | . | . | . |
|  | JM/BID-V2963/2007 | . | . | . | . | S | . | . | . | . | . | . | I | . | . | . |
|  | US/BID-V1439/2005 | . | . | . | . | . | . | . | . | . | . | N | . | . | . | . |
|  | VI/BID-V2960/2005 | . | . | . | . | S | . | . | . | . | . | . | . | . | . | . |
| BR2 | **BR/BID-V2377/2000** | V | . | . | . | . | . | . | . | . | . | . | . | . | . | V |
| BR2 | **BR/BID-V2390/2004** | V | . | . | . | . | V | . | . | . | . | . | . | . | . | V |
| BR2 | **BR/BID-V2393/2005** | V | . | . | . | . | V | . | . | . | . | . | . | . | . | V |
| BR2 | **BR/BID-V2382/2002** | V | . | P | . | . | . | . | . | . | . | . | . | . | . | V |
| BR2 | **BR/BID-V2385/2003** | V | . | P | . | . | . | . | . | . | . | . | . | . | . | V |
| BR2 | **BR/BID-V2379/2001** | V | . | P | . | . | . | . | . | . | . | . | . | . | . | V |
| BR2 | **BR/BID-V2386/2003** | V | . | P | A | . | . | . | . | . | . | . | . | . | . | V |
| BR2 | **BR/BID-V2396/2006** | V | . | P | A | . | . | . | . | . | . | . | . | . | . | V |
|  | US/BID-V678/1998 | V | . | . | . | . | . | . | . | . | . | . | . | . | . | V |
|  | US/BID-V1032/1998 | V | . | . | . | . | . | . | . | . | . | . | . | . | . | V |
| BR1 | **BR64022** | V | . | . | . | . | . | . | I | Y | . | . | . | V | . | V |
| BR1 | **BR/BID-V2376/2000** | V | . | . | . | . | . | . | . | . | . | . | . | . | . | V |
|  | VE/BID-V1456/1996 | V | . | . | . | S | . | . | . | . | . | . | . | . | . | V |
|  | VE/BID-V2941/1998 | V | . | . | . | S | . | . | . | . | . | . | . | . | M | V |

Table S3. Amino acids substitutions observed in the whole deduced sequence of DENV-2, characterizing different lineages of Dengue virus 2 from Brazil (continued).

| L | Strains | Protein and amino acid position | | | | | | | | | | | | | | | |  |
| --- | --- | --- | --- | --- | --- | --- | --- | --- | --- | --- | --- | --- | --- | --- | --- | --- | --- | --- |
|  |  | NS2a | | | | | | | | | | | | | | | |  |
|  |  | 40 | 62 | 63 | 64 | 108 | 109 | 116 | 133 | 136 | 138 | 142 | 151 | 159 | 162 | 174 | 189 |  |
| BR3 | **BR/BID-V2402/2008** | F | A | T | M | I | A | L | A | I | R | K | A | V | Q | A | A | |
| BR3 | **BR/BID-V2399/2007** | . | . | . | . | . | . | . | . | . | . | . | . | . | . | . | . |  |
| BR3 | ***BR/BID-V3644/2008*** | . | . | . | . | . | . | . | . | . | . | . | . | . | . | . | . |  |
| BR3 | ***BR/BID-V3648/2008*** | . | . | . | . | . | . | . | . | . | . | . | . | . | . | . | . |  |
| BR3 | ***BR/BID-V3650/2008*** | . | . | . | . | . | . | . | . | . | . | . | . | . | . | . | . |  |
| BR3 | ***BR/BID-V3653/2008**** | . | . | . | . | . | . | . | . | . | . | . | . | . | . | . | . |  |
| BR3 | ***BR/BID-V3481/2008*** | . | . | . | . | . | . | . | . | . | . | . | . | . | . | . | . |  |
| BR3 | ***BR/BID-V3495/2008*** | . | . | . | . | . | . | . | . | . | . | . | . | . | . | . | . |  |
| BR3 | ***BR/BID-V3645/2008*** | . | . | . | . | . | . | . | . | . | . | . | . | . | . | . | . |  |
| BR3 | ***BR/BID-V3486/2008*** | . | . | . | . | . | . | . | . | . | . | . | . | . | . | . | . |  |
| BR3 | ***BR/BID-V3637/2008*** | . | . | . | . | . | . | . | . | . | . | . | . | . | . | . | . |  |
|  | JM/BID-V2963/2007 | . | . | . | . | . | . | . | . | . | . | . | . | . | . | . | . |  |
|  | US/BID-V1439/2005 | . | . | . | . | . | . | . | V | . | . | . | . | . | . | . | . |  |
|  | VI/BID-V2960/2005 | . | . | . | . | . | . | . | V | . | . | . | . | . | . | . | . |  |
| BR2 | **BR/BID-V2377/2000** | . | . | . | . | V | . | I | . | . | K | . | . | . | . | . | . |  |
| BR2 | **BR/BID-V2390/2004** | . | . | . | . | V | . | I | . | . | K | . | . | . | . | . | . |  |
| BR2 | **BR/BID-V2393/2005** | . | . | . | . | V | . | I | . | . | K | . | . | . | . | . | . |  |
| BR2 | **BR/BID-V2382/2002** | . | . | . | . | V | . | I | . | . | K | . | . | . | . | . | . |  |
| BR2 | **BR/BID-V2385/2003** | . | . | . | I | V | . | I | . | . | K | . | . | . | . | . | . |  |
| BR2 | **BR/BID-V2379/2001** | . | . | . | . | V | . | I | . | . | K | . | . | . | . | . | . |  |
| BR2 | **BR/BID-V2386/2003** | . | . | . | . | V | . | I | . | . | K | . | . | . | . | . | . |  |
| BR2 | **BR/BID-V2396/2006** | L | . | . | . | V | . | I | . | V | K | . | . | . | . | . | . |  |
|  | US/BID-V678/1998 | . | . | . | . | . | . | I | . | . | K | . | . | . | . | . | . |  |
|  | US/BID-V1032/1998 | . | . | . | . | . | . | I | . | . | K | . | . | . | . | . | . |  |
| BR1 | **BR64022** | . | V | . | . | . | . | I | V | . | . | . | . | . | L | . | T |  |
| BR1 | **BR/BID-V2376/2000** | . | V | A | . | . | T | I | V | . | . | . | T | M | L | V | T |  |
|  | VE/BID-V1456/1996 | . | . | . | . | . | . | I | V | . | . | . | . | . | L | . | . |  |
|  | VE/BID-V2941/1998 | . | . | . | . | . | . | I | V | . | . | N | . | . | L | . | . |  |

Table S3. Amino acids substitutions observed in the whole deduced sequence of DENV-2, characterizing different lineages of Dengue virus 2 from Brazil (continued).

| L | Strains | Protein and amino acid position | | | | | | | | | | | | | | | |
| --- | --- | --- | --- | --- | --- | --- | --- | --- | --- | --- | --- | --- | --- | --- | --- | --- | --- |
|  |  | NS3 | | | | | | | | | | | | | | | |
|  |  | 14 | 20 | 77 | 115 | 122 | 169 | 182 | 249 | 395 | 399 | 418 | 461 | 466 | 535 | 549 | 561 |
| BR3 | **BR/BID-V2402/2008** | G | D | I | I | T | E | I | A | V | A | K | I | K | D | K | K |
| BR3 | **BR/BID-V2399/2007** | . | . | . | . | . | . | . | . | A | . | . | . | . | . | . | . |
| BR3 | ***BR/BID-V3644/2008*** | . | . | . | . | . | . | . | . | . | . | . | . | . | . | . | . |
| BR3 | ***BR/BID-V3648/2008*** | . | . | . | . | . | . | . | . | . | . | . | . | . | . | . | . |
| BR3 | ***BR/BID-V3650/2008*** | . | . | . | . | . | . | . | . | . | . | . | . | . | . | . | . |
| BR3 | ***BR/BID-V3653/2008**** | . | . | . | . | . | . | . | . | . | . | . | . | . | . | . | . |
| BR3 | ***BR/BID-V3481/2008*** | . | . | . | . | . | . | . | . | . | . | . | . | . | . | . | . |
| BR3 | ***BR/BID-V3495/2008*** | . | . | . | . | . | . | . | . | . | . | . | . | . | . | . | . |
| BR3 | ***BR/BID-V3645/2008*** | . | . | . | . | . | . | . | . | . | . | . | . | . | . | . | . |
| BR3 | ***BR/BID-V3486/2008*** | . | . | . | . | . | . | . | . | . | . | . | . | . | . | . | . |
| BR3 | ***BR/BID-V3637/2008*** | . | . | . | . | . | . | . | . | . | . | . | . | . | . | . | . |
|  | JM/BID-V2963/2007 | . | . | . | . | . | . | . | . | . | . | . | . | . | . | . | . |
|  | US/BID-V1439/2005 | . | . | . | . | . | . | . | . | . | . | . | . | . | E | . | . |
|  | VI/BID-V2960/2005 | . | . | . | . | . | . | . | . | . | . | . | . | . | . | . | . |
| BR2 | **BR/BID-V2377/2000** | . | . | V | . | N | . | . | . | . | . | . | . | . | . | . | R |
| BR2 | **BR/BID-V2390/2004** | . | E | V | . | . | D | . | . | . | . | . | . | . | . | . | R |
| BR2 | **BR/BID-V2393/2005** | . | E | V | . | . | D | . | . | . | . | . | . | . | . | . | R |
| BR2 | **BR/BID-V2382/2002** | . | . | V | . | . | . | . | . | . | S | . | . | . | . | . | R |
| BR2 | **BR/BID-V2385/2003** | . | . | V | . | . | . | . | . | . | S | . | . | . | . | . | R |
| BR2 | **BR/BID-V2379/2001** | . | . | V | . | . | . | . | . | . | S | . | . | . | . | . | R |
| BR2 | **BR/BID-V2386/2003** | E | . | V | . | . | . | . | . | . | S | . | . | . | . | . | R |
| BR2 | **BR/BID-V2396/2006** | E | . | V | . | . | . | . | . | . | S | . | . | . | . | . | R |
|  | US/BID-V678/1998 | . | . | V | . | . | . | . | . | . | . | . | . | . | . | . | R |
|  | US/BID-V1032/1998 | . | . | V | . | . | . | . | . | . | . | . | . | . | . | . | R |
| BR1 | **BR64022** | E | . | . | L | . | . | F | T | . | T | R | . | R | . | R | R |
| BR1 | **BR/BID-V2376/2000** | E | . | . | L | . | . | . | . | . | . | R | V | . | . | R | R |
|  | VE/BID-V1456/1996 | . | . | . | L | . | . | . | . | . | . | R | . | . | . | R | R |
|  | VE/BID-V2941/1998 | . | . | . | L | . | . | . | . | . | . | R | . | . | . | R | R |

Table S3. Amino acids substitutions observed in the whole deduced sequence of DENV-2, characterizing different lineages of Dengue virus 2 from Brazil (continued).

| L | Strains | Protein and amino acid position | | | | | | | | | | | | | | |
| --- | --- | --- | --- | --- | --- | --- | --- | --- | --- | --- | --- | --- | --- | --- | --- | --- |
|  |  | NS2b | | | | | | | NS4a | | | | | | | |
|  |  | 8 | 26 | 48 | 57 | 64 | 87 | 116 | 23 | 36 | 39 | 42 | 59 | 63 | 64 | 78 |
| BR3 | **BR/BID-V2402/2008** | I | I | S | A | Q | K | I | N | V | K | T | T | T | I | I |
| BR3 | **BR/BID-V2399/2007** | . | . | A | . | . | . | . | . | . | . | . | . | A | V | . |
| BR3 | ***BR/BID-V3644/2008*** | . | . | . | . | . | . | . | . | . | . | . | . | A | V | . |
| BR3 | ***BR/BID-V3648/2008*** | . | . | . | . | . | . | . | . | . | . | . | . | A | V | . |
| BR3 | ***BR/BID-V3650/2008*** | . | . | . | . | . | . | . | . | . | . | . | . | A | V | . |
| BR3 | ***BR/BID-V3653/2008**** | . | . | . | . | . | . | . | . | . | . | . | . | A | V | . |
| BR3 | ***BR/BID-V3481/2008*** | . | . | . | . | . | . | . | . | . | . | . | . | A | V | . |
| BR3 | ***BR/BID-V3495/2008*** | . | . | . | . | . | . | . | . | . | . | . | . | A | V | . |
| BR3 | ***BR/BID-V3645/2008*** | . | . | . | . | . | . | . | . | . | . | . | . | A | V | . |
| BR3 | ***BR/BID-V3486/2008*** | . | . | . | . | . | . | . | . | . | . | . | . | A | V | . |
| BR3 | ***BR/BID-V3637/2008*** | . | . | . | . | . | . | . | . | . | . | . | . | A | V | . |
|  | JM/BID-V2963/2007 | . | . | . | . | . | . | . | . | . | R | . | . | . | V | . |
|  | US/BID-V1439/2005 | . | . | . | . | . | . | . | . | . | R | N | . | . | V | V |
|  | VI/BID-V2960/2005 | . | . | . | . | . | . | . | . | . | R | N | . | . | V | . |
| BR2 | **BR/BID-V2377/2000** | . | . | . | . | . | . | . | . | A | . | N | . | . | V | . |
| BR2 | **BR/BID-V2390/2004** | . | . | . | . | . | . | . | . | A | . | N | . | . | V | . |
| BR2 | **BR/BID-V2393/2005** | . | . | . | . | . | . | . | . | A | . | N | . | . | V | . |
| BR2 | **BR/BID-V2382/2002** | . | V | . | . | . | . | . | . | A | . | N | . | . | V | . |
| BR2 | **BR/BID-V2385/2003** | . | V | . | . | . | . | . | . | A | . | N | . | . | V | . |
| BR2 | **BR/BID-V2379/2001** | . | . | . | . | . | . | . | . | A | . | N | . | . | V | . |
| BR2 | **BR/BID-V2386/2003** | V | . | . | . | . | . | . | . | A | . | N | . | . | V | . |
| BR2 | **BR/BID-V2396/2006** | V | . | . | . | . | . | . | . | A | . | N | A | . | V | . |
|  | US/BID-V678/1998 | . | . | . | . | . | R | V | . | A | . | N | . | . | V | . |
|  | US/BID-V1032/1998 | . | . | . | . | . | R | V | . | A | . | N | . | . | V | . |
| BR1 | **BR64022** | V | . | . | . | . | . | . | D | A | R | N | . | . | V | . |
| BR1 | **BR/BID-V2376/2000** | V | . | . | T | . | . | . | . | A | R | N | . | . | V | . |
|  | VE/BID-V1456/1996 | V | . | . | . | H | . | . | D | A | R | N | . | . | V | . |
|  | VE/BID-V2941/1998 | V | . | . | . | H | . | . | D | A | R | N | . | . | V | . |

Table S3. Amino acids substitutions observed in the whole deduced sequence of DENV-2, characterizing different lineages of Dengue virus 2 from Brazil (continued).

| L | Strains | Protein and amino acid position | | | | | | | | | | | | | | | | | |
| --- | --- | --- | --- | --- | --- | --- | --- | --- | --- | --- | --- | --- | --- | --- | --- | --- | --- | --- | --- |
|  |  | NS4b | | | | | | | NS5 | | | | | | | | | | |
|  |  | 15 | 17 | 48 | 91 | 113 | 183 | 239 | 5 | 10 | 23 | 30 | 105 | 168 | 200 | 271 | 341 | 375 | 388 |
| BR3 | **BR/BID-V2402/2008** | F | T | V | V | L | M | I | V | A | S | K | K | V | K | I | M | K | E |
| BR3 | **BR/BID-V2399/2007** | . | S | . | . | . | . | . | . | . | . | . | . | . | . | . | . | . | . |
| BR3 | ***BR/BID-V3644/2008*** | . | S | . | . | . | . | . | . | . | . | . | . | . | . | . | . | . | . |
| BR3 | ***BR/BID-V3648/2008*** | . | S | . | . | . | . | . | . | . | . | . | . | . | . | . | . | . | . |
| BR3 | ***BR/BID-V3650/2008*** | . | S | . | . | . | . | . | . | . | . | . | . | . | . | . | . | . | . |
| BR3 | ***BR/BID-V3653/2008**** | . | S | . | . | . | . | . | . | . | . | . | . | . | . | . | . | . | . |
| BR3 | ***BR/BID-V3481/2008*** | . | S | . | . | . | . | . | . | . | . | . | . | . | . | . | . | . | . |
| BR3 | ***BR/BID-V3495/2008*** | . | S | . | . | . | . | . | . | . | . | . | . | . | . | . | . | . | . |
| BR3 | ***BR/BID-V3645/2008*** | . | S | . | . | . | . | . | . | . | . | . | . | . | . | . | . | . | . |
| BR3 | ***BR/BID-V3486/2008*** | . | S | . | . | . | . | . | . | . | . | . | . | . | . | . | . | . | . |
| BR3 | ***BR/BID-V3637/2008*** | . | S | . | . | . | . | . | . | . | . | . | . | . | . | . | . | . | . |
|  | JM/BID-V2963/2007 | . | S | . | . | . | . | . | . | . | . | . | . | . | . | . | . | . | . |
|  | US/BID-V1439/2005 | . | S | I | . | . | . | . | . | . | . | . | . | . | . | . | . | . | . |
|  | VI/BID-V2960/2005 | . | S | . | . | . | . | . | . | . | . | . | . | . | . | . | . | . | . |
| BR2 | **BR/BID-V2377/2000** | L | S | . | A | . | . | . | I | . | . | . | . | . | . | . | . | . | K |
| BR2 | **BR/BID-V2390/2004** | L | S | . | . | . | . | . | I | . | . | . | . | . | . | V | . | . | K |
| BR2 | **BR/BID-V2393/2005** | L | S | . | . | . | . | . | I | . | . | . | . | . | . | V | . | . | K |
| BR2 | **BR/BID-V2382/2002** | L | S | . | . | . | . | . | I | . | . | . | . | . | . | . | . | . | K |
| BR2 | **BR/BID-V2385/2003** | L | S | . | . | . | . | . | I | . | . | . | . | . | . | . | . | . | K |
| BR2 | **BR/BID-V2379/2001** | L | S | . | . | . | . | . | I | . | . | . | . | . | . | . | . | . | K |
| BR2 | **BR/BID-V2386/2003** | L | S | . | . | . | . | . | I | . | . | . | R | . | . | . | . | . | K |
| BR2 | **BR/BID-V2396/2006** | L | S | . | . | . | . | V | I | . | . | . | R | . | T | . | . | . | K |
|  | US/BID-V678/1998 | L | S | . | . | . | T | . | I | . | . | . | . | . | . | . | . | . | K |
|  | US/BID-V1032/1998 | L | S | . | . | . | . | . | I | . | . | . | . | . | . | . | . | . | K |
| BR1 | **BR64022** | L | S | . | . | . | . | . | I | T | N | . | . | A | . | . | V | R | K |
| BR1 | **BR/BID-V2376/2000** | L | S | I | . | S | . | . | I | . | . | R | . | . | . | . | . | R | K |
|  | VE/BID-V1456/1996 | L | S | . | . | . | . | . | I | . | . | . | . | . | . | . | . | R | K |
|  | VE/BID-V2941/1998 | L | S | . | . | . | . | . | I | . | . | . | . | . | . | . | . | R | K |

Table S3. Amino acids substitutions observed in the whole deduced sequence of DENV-2, characterizing different lineages of Dengue virus 2 from Brazil (continued).

| L | Strains | Protein and amino acid position | | | | | | | | | | | | | | | | | | |
| --- | --- | --- | --- | --- | --- | --- | --- | --- | --- | --- | --- | --- | --- | --- | --- | --- | --- | --- | --- | --- |
|  |  | NS5 | | | | | | | | | | | | | | | | | | |
|  |  | 390 | 412 | 429 | 501 | 521 | 523 | 524 | 553 | 568 | 596 | 637 | 648 | 656 | 670 | 676 | 723 | 874 | 878 | 891 |
| BR3 | **BR/BID-V2402/2008** | T | I | G | G | E | G | K | V | I | K | A | A | S | I | S | V | I | G | K |
| BR3 | **BR/BID-V2399/2007** | . | V | . | . | . | . | . | . | . | . | . | . | . | . | . | . | . | . | R |
| BR3 | ***BR/BID-V3644/2008*** | . | V | . | . | . | . | . | . | . | . | . | . | . | . | . | . | . | . | R |
| BR3 | ***BR/BID-V3648/2008*** | . | V | . | . | . | . | . | . | . | . | . | . | . | . | . | . | M | . | R |
| BR3 | ***BR/BID-V3650/2008*** | . | V | . | . | . | . | . | . | . | . | . | . | . | . | . | . | M | . | R |
| BR3 | ***BR/BID-V3653/2008**** | . | V | . | . | . | . | . | . | . | . | . | . | . | . | . | . | M | . | R |
| BR3 | ***BR/BID-V3481/2008*** | . | V | . | . | . | . | . | . | . | . | . | . | . | . | . | . | M | . | R |
| BR3 | ***BR/BID-V3495/2008*** | . | V | . | . | . | . | . | . | . | . | . | . | . | . | . | . | M | . | R |
| BR3 | ***BR/BID-V3645/2008*** | . | V | . | . | . | . | . | . | . | . | . | . | . | . | . | . | M | . | R |
| BR3 | ***BR/BID-V3486/2008*** | . | V | . | . | . | . | . | . | . | . | . | . | . | . | . | . | M | . | R |
| BR3 | ***BR/BID-V3637/2008*** | . | V | . | . | . | . | . | . | . | . | . | . | . | . | . | . | M | . | R |
|  | JM/BID-V2963/2007 | . | . | . | . | . | . | . | . | . | . | . | . | . | . | . | . | . | . | R |
|  | US/BID-V1439/2005 | . | . | . | . | . | . | . | . | . | . | . | . | . | . | . | . | . | E | R |
|  | VI/BID-V2960/2005 | . | . | . | . | . | . | . | . | . | . | V | . | . | . | . | . | . | E | R |
| BR2 | **BR/BID-V2377/2000** | I | . | . | . | D | S | . | . | . | R | V | . | T | . | N | . | . | E | R |
| BR2 | **BR/BID-V2390/2004** | I | . | . | . | D | S | . | I | . | R | V | . | T | . | N | . | . | . | R |
| BR2 | **BR/BID-V2393/2005** | I | . | . | . | D | S | . | I | . | R | V | . | T | . | N | . | . | . | R |
| BR2 | **BR/BID-V2382/2002** | . | . | . | . | D | S | . | . | . | R | V | . | T | . | N | . | . | E | R |
| BR2 | **BR/BID-V2385/2003** | . | . | . | . | D | S | . | . | . | R | V | . | T | . | N | . | . | E | R |
| BR2 | **BR/BID-V2379/2001** | . | . | . | . | D | S | . | . | . | R | V | . | T | . | N | . | . | E | R |
| BR2 | **BR/BID-V2386/2003** | . | . | . | . | D | S | . | . | . | R | V | . | T | . | N | . | . | E | R |
| BR2 | **BR/BID-V2396/2006** | . | . | . | . | D | S | R | . | . | R | V | . | T | . | N | . | . | E | R |
|  | US/BID-V678/1998 | . | . | . | . | D | S | . | . | . | R | V | . | . | . | . | . | . | E | R |
|  | US/BID-V1032/1998 | . | . | . | . | D | S | . | . | . | R | V | . | . | . | . | . | . | E | R |
| BR1 | **BR64022** | . | . | S | E | D | S | . | . | . | R | V | . | . | L | . | . | . | E | R |
| BR1 | **BR/BID-V2376/2000** | . | . | S | . | D | S | . | . | V | R | V | . | . | L | . | M | . | E | R |
|  | VE/BID-V1456/1996 | . | . | S | . | D | S | . | . | . | R | V | V | . | L | . | . | . | E | R |
|  | VE/BID-V2941/1998 | . | . | S | . | D | S | . | . | . | R | V | . | . | L | . | . | . | E | R |

L: Lineages. Brazilian isolates are shown in bold and strains from São José do Rio Preto/São Paulo are in bold and italic. Dots represent identical amino acid. (*) BR/BID-V3638/2008, BR/BID-V3640/2008 and BR/BID-V3483/2008 are identical to BR/BID-V3653/2008. Isolates from Jamaica (JM/BID-V2963/2007), Puerto Rico (US/BID-V1439/2005, US/BID-V678/1998, US/BID-V1032/1998), The Virgin Islands (VI/BID-V2960/2005) and Venezuela (VE/BID-V1456/1996, VE/BID-V2941/1998) are also included in the table.
