# Supplementary material for: Circulation of Different Lineages of Dengue Virus 2, Genotype American/Asian in Brazil: Dynamics and Molecular and Phylogenetic Characterization
Source: PLoS One. 2013 Mar 22;8(3):e59422. doi: 10.1371/journal.pone.0059422 (PMC3606110; doi:10.1371/journal.pone.0059422)
Supplement: Table S4 — Estimate of Most Recent Common Ancestor (MRCA) of Brazilian DENV-2 lineages, based on envelope gene sequence. (DOC) [file pone.0059422.s004.doc]

**Table S4.** Estimate of Most Recent Common Ancestor (MRCA) of Brazilian DENV-2 lineages, based on envelope gene sequence.

| Lineages | Relaxed clock | | Strict Clock | |
| --- | --- | --- | --- | --- |
|  | MRCA ± SE * (MRCA 95% HPD*) | Estimated year (years 95% HPD) | MRCA ± SE * (MRCA 95% HPD*) | Estimated year (years 95% HPD) |
| BR1 | 20.94* ± 0.002 (20.30– 21.67*) | 1989 (1988-1989) | 21,06* ± 0.004 (20,35 - 21,81*) | 1989 (1988-1989) |
| BR2 | 11.00* ± 0.003 (10.17– 11.92*) | 1999 (1998-2000) | 10,95* ± 0.005 (10,18 - 11,77*) | 1999 (1998-2000) |
| BR3^#^ | 5.74* ± 0.004 (4.68 – 6.97*) | 2004 (2003-2005) | 6.15* ± 0.008 (4,98-7,44*) | 2004 (2003-2005) |

Mean values from three independent runs are presented here. *years before 2010. ^#^ Strain JM-BID-V2963/2007 was included in lineage BR3 in this analysis. MRCA: most recent common ancestor; SE: standard error, 95% HPD: highest probability density intervals. Coalescent analyses were performed using programs from BEAST package v.1.6.1, BEAUTi, Tracer v.1.5.0, TreeAnotator v.1.6.1 and FigTree v.1.3.1.
